# The Cellular and Biological Impact of Extracellular Vesicles in Pancreatic Cancer

**DOI:** 10.3390/cancers13123040

**Published:** 2021-06-18

**Authors:** Zainab Hussain, Jeremy Nigri, Richard Tomasini

**Affiliations:** INSERM, U1068, Cancer Research Center of Marseille, Institut Paoli-Calmettes, CNRS, UMR7258, University Aix-Marseille, 13009 Marseille, France; zainab.hussain@inserm.fr (Z.H.); jeremy.nigri@inserm.fr (J.N.)

**Keywords:** extracellular vesicles, pancreatic cancer, stromal cells, biological impact

## Abstract

**Simple Summary:**

The increased incidence and global failure of ongoing therapies project pancreatic cancer as the second deadliest cancer worldwide. While our knowledge of pancreatic cancer cells’ abilities and specificities has drastically improved based on multi-scaled omics, one must consider that much more remains to be uncovered on the role and impact of stromal cells and the established network of communication with tumor cells. This review article discusses how tumor cells communicate with the various cells composing the stroma and its implication in tumor cells’ abilities, PDA (pancreatic ductal adenocarcinoma) carcinogenesis and therapeutic response. We will focus on extracellular vesicles-mediated crosstalk and how this multifaceted dialogue impacts both cellular compartments and its subsequent impact on PDA biology.

**Abstract:**

Deciphering the interactions between tumor and stromal cells is a growing field of research to improve pancreatic cancer-associated therapies and patients’ care. Indeed, while accounting for 50 to 90% of the tumor mass, many pieces of evidence reported that beyond their structural role, the non-tumoral cells composing the intra-tumoral microenvironment influence tumor cells’ proliferation, metabolism, cell death and resistance to therapies, among others. Simultaneously, tumor cells can influence non-tumoral neighboring or distant cells in order to shape a tumor-supportive and immunosuppressive environment as well as influencing the formation of metastatic niches. Among intercellular modes of communication, extracellular vesicles can simultaneously transfer the largest variety of signals and were recently reported as key effectors of cell–cell communication in pancreatic cancer, from its development to its evolution as well as its ability to resist available treatments. This review focuses on extracellular vesicles-mediated communication between different cellular components of pancreatic tumors, from the modulation of cellular activities and abilities to their biological and physiological relevance. Taking into consideration the intra-tumoral microenvironment and its extracellular-mediated crosstalk as main drivers of pancreatic cancer development should open up new therapeutic windows.

## 1. Introduction

Pancreatic cancer (mainly pancreatic ductal adenocarcinoma, PDA) represents the fourth leading cause of cancer-related death in western countries and is projected to become the second by 2030 [1]. With an overall 5-year survival rate at only ~8% and a median survival rate of 6 months if left untreated [2,3], together with an unexplained increased incidence [4], pancreatic cancer figures as one of the solid cancers with the worst prognosis. Such bleak epidemiological data have forced institutions to support the scientific community in expanding our knowledge of PDA. The main reasons as to why survival statistics have hardly changed in the past 20 years include a late diagnosis, a rapid progression towards advancing stages of the disease, a lack of biomarkers and the prevalence of therapeutic resistance. Indeed, while surgical resection remains our best therapeutic option, only ~20% of patients are diagnosed with a resectable PDA. The lack of early symptoms combined with the absence of specific and relevant biomarkers lead to this dramatic outcome in which the remaining therapeutic options are the use of combinatorial chemotherapies with important side effects and rather low response rates. Thus, clinicians are confronted with advanced and aggressive tumors with few therapeutic options, highlighting the urgent need to better understand the molecular mechanisms underlying pancreatic cancer carcinogenesis and therapeutic resistance in order to design new clinical tools to improve patients’ management.

As little as 10 years ago, the scientific community began to evaluate the tumor microenvironment (TME) as a putative active member of pancreatic cancer development and evolution rather than just a supportive or structural tissue. This former vision of PDA led to the substantial development of therapeutic tools focused on cancer cell targeting, neglecting the impact of the stromal compartment, which could explain the rather limited improvement in patients’ survival over the last 20 years. At present, while this statement was entirely revised, the recent progress of our knowledge on the impact of TME, and the associated clinical trials targeting this cellular compartment, have not yet led to better patient management. This suggests that much more is needed to be discovered on the functional relationship between the various cell types composing PDA before integrating and subsequently transforming this knowledge into clinical tools to improve patient care. It is now well established that PDA can be composed of up to 90% of stromal cells, mainly cancer-associated fibroblasts (CAFs), and a variety of immune cells as well as endothelial and nerve cells. Numerous studies revealed the key roles of these non-tumoral cells in PDA development [5]. Indeed, the TME was recently reported to shape PDA cancer cell heterogeneity [6] but can also modulate PDA immunity [7] and metabolic reprogramming [8], among many other processes. However, the global picture of such an intricate network of cells composing PDA, between close and even more distal neighbors, and the importance of their mode of communication is still misunderstood. Their potential as sources of new therapeutic and biomarker options remains unchallenged and should open a promising area of clinical improvements.

Among intercellular modes of communication, accumulating evidence represents extracellular vesicles (EVs) as cargos able to simultaneously transfer the largest variety of signals such as proteins, metabolites, lipids or nucleic acids. They are referenced under several subtypes (i.e., exosomes, microvesicles, apoptotic cell-derived exosomes) depending on their size, cargo composition, cells of origin or biogenesis [9]. Besides the complexity of the transmitted message, EVs-mediated crosstalk is further regulated by their mode of action or internalization in recipient cells, including macropinocytosis, receptor-mediated endocytosis, phagocytosis or receptor-mediated signaling, which influences the ultimate fate of the transmitted information [10]. EVs are now reported as major actors of cellular crosstalk in physiological and physio-pathological contexts involving adaptive and responsive processes [11], which suggests that EVs’ production, secretion and uptake are part of a larger network of communication.

In PDA, several studies reported EVs as playing important roles in tumor cell aggressiveness, niche establishment and chemoresistance, among others, as well as in many stromal cell-associated functions. The prevalence of stromal cells in PDA further reinforces the potential role of EVs and intercellular communication in its physiopathology. In this review, we will discuss key concepts surrounding the current understanding of the role of EVs in intercellular crosstalk in PDA, from its molecular mechanisms to its cellular and biological impacts.

## 2. Tumor Cells EVs-Mediated Crosstalk

During the past decade, PDA has been characterized as an intra- and inter-patient heterogeneous cancer. Several studies stratified PDA in molecular subtypes regarding their genetic alterations or expression profiles [12]. Mainly named as classical, basal and immunogenic, these molecular stratifications were further associated with patients’ survival or therapeutic response [13]. However, besides these various classifications, PDA is further characterized by an important intra-tumoral heterogeneity, which reflects not only the presence of several tumoral clones but also the simultaneous presence of tumor cells from several molecular subtypes in a single tumor, and even in a single tumoral gland structure [6]. This heterogeneity is further pronounced by the environmental context of these tumor cells that can be either hypoxic, enriched in extracellular matrix (ECM) components or clustered by a shield of stromal cells, among others. Hence, the communication between tumor cells of different subtypes, clones, localization or subjected to varying microenvironments further complexify the potential cellular network established in each tumor. Understanding how tumor cells influence their own capacities is an important challenge to address in order to avoid tumor progression and resistance to treatments (Table 1).

### 2.1. Tumor Progression

Recent discoveries identified that PDA cells transfer biological messengers in the form of extracellular vesicles among themselves to promote tumor progression and invasion, where these EVs are found to be secreted by advantaged, invasive or tumor-initiating cells. It is important to emphasize that studies investigating this crosstalk have consistently focused on specific messengers carried by EVs. In the light of these findings, in the following section, we will describe the implication of microRNAs, long non-coding RNAs and specific cancer stem cell markers, as well as proteins and lipids transferred by EVs and implicated in promoting PDA cancer cell aggressiveness and tumor progression.

#### 2.1.1. MicroRNAs

MicroRNAs (miRNAs) are small non-coding RNA molecules found in all human cells. miRNAs negatively regulate gene expression by hybridizing in a base-pairing manner to its target mRNAs, inhibiting their translation [14]. While the control of mRNA translation by miRNAs occurs in specific cytoplasmic granules called mRNA processing bodies (P-bodies), the specificity of action of miRNAs is still underestimated as a single miRNA that can target several mRNAs and that, for most miRNAs, their cellular targets and function are yet unknown. It is important to note that miRNAs are stable within EVs as their degradation by RNAse enzymes is prevented [15]. In pancreatic cancer, miRNAs were found to be associated with tumor-specific mRNA subtypes [16], while the expression of several tumor-suppressive [17] or oncogenic [18] miRNAs was correlated with PDA patients’ survival. Recently, miRNAs were broadly associated with key intermediates of biological pathways in pancreatic cancers [19] but also investigated as relevant therapeutic options [20].

Several miRNAs involved in tumor evolution were brought to light as key biomarkers in EVs for diagnostic purposes. Indeed, Zou et al. reported the identification of a panel of six miRNAs in a serum benefiting pancreatic cancer diagnosis [21]. Zhou et al. evaluated plasma miRNAs in the diagnosis and prognosis of PDA [22], while Vincentini et al. revealed an exosomal miRNA signature associated with tumoral pancreatic lesions [23]. Although these studies identified such miRNAs as potential biomarkers, the cellular origins of these miRNA-carrying EVs remain unknown. A recent study from Zeöld et al. confirmed this miRNA profiling by establishing shared extracellular vesicles’ miRNA profiles of matched PDA organoids and patient blood plasma samples, suggesting PDA tumor cells as cells of origin [24]. Specifically designed studies of tumor cell-derived EVs further reported the prognostic value of the tumor-suppressive miRNA, miR-192-5p [25].

Aside from the use of miRNAs in circulating EVs and tumor cell-derived EVs as key biomarkers in PDA, recent studies are now unveiling miRNAs- and EV-associated tumorigenic mechanisms. Chen et al. reported that miR-23b-3p, upregulated in PDA as well as renal, gastric and non-small cell lung cancers, was closely associated with CA-19-9 levels, the traditional clinical biomarker for PDA diagnosis and promoted proliferation, invasion and migration of classic PDA cell lines [26]. More recently, Yu et al. studied the role of miRNA-339-5p in exosomes of a highly metastatic PDA cell line, demonstrating increased invasion and migration. miRNA-339-5p levels were significantly lower than in classic tumor cells, hypothetically through an effect on its target, zinc-finger protein ZNF689 known to promote tumor progression [27]. Such impact of tumor cell-derived exosomal-miRNA on migration and invasion is further strengthened by a report from Sonohara et al. highlighting the association with epithelial-to-mesenchymal transition (EMT) [28]. In contrast to the above studies, upregulated and functional miR-222 transfer from highly invasive to non-invasive cells via exosomes was experimentally confirmed, promoting cell invasion and proliferation in recipient cells through subsequent phosphorylation and activation of Akt, relocalizing p27 to the cytoplasm, thus suppressing its capacity as a tumor suppressor in the nucleus [29]. The impact of miRNAs on pro-metastatic profiles of PDA tumor cells was further reinforced by Wu et al. Their study demonstrated that metastatic PDA tumor cells-derived EVs confer pro-metastatic abilities to non-metastatic PDA tumor cells. This gain of function and associated phenotype is related to the transfer of miRNA-125b-5p, which represses the tumor suppressor STARD13, increasing the MEK2/ERK pathway [30]. Investigating the pro-metastatic role of metastatic PDA tumor cells-derived EVs and the associated aberrant expression of exosomal miRNAs may help to elucidate the metastatic mechanism of pancreatic cancer.

#### 2.1.2. Long Non-Coding RNAs (LncRNAs)

In addition to miRNAs, the genome contains other types of non-coding RNAs, such as the long non-coding RNAs (lncRNAs). Greater than 200 nucleotides and generated from various genetic DNA elements including enhancers, intergenic sites and promoters, the implication of lncRNAs in regulating gene expression at the chromatin, DNA, mRNA and miRNA levels was clearly demonstrated even if the specific production and regulation mechanisms of some lncRNAs remain unclear. Interestingly, while lncRNAs are known to play key roles during differentiation and development in humans, lncRNAs are aberrantly expressed in a wide variety of cancers. The dysregulation of lncRNAs was even proposed to be a hallmark property of malignancies [31]. Determining their impact in EVs-receiving cells is important as lncRNAs account for about 20% of total EVs’ RNA content in certain cancers and are shown to regulate global cell features such as apoptosis, invasive capacity and eventually metastases.

Similar to miRNAs, lncRNAs’ profiles in circulating EVs of PDA patients are being investigated to highlight potential biomarkers [32]. Li et al., using plasma-derived EVs from PDA patients, revealed a specific lncRNA, Sox2 overlapping transcript (Sox2ot), as correlated with aggressive clinical features and lower survival. At the mechanistic level, Sox2ot transferred from PDA tumor cells-derived EVs into PDA tumor cells increased mesenchymal marker expression, indicative of EMT, as well as cancer stem cell marker expression. This results from the competitive endogenous RNA (ceRNA) activity of lncRNA Sox2ot with miRNA 200, lifting the repression of Soxo2 expression and thus leading to EMT and stem cell features. EVs-receiving PDA cells with these features further demonstrated increased invasion and metastases in vivo, presumably since EMT and stem features facilitate dissemination and seeding in secondary sites [33]. The comparison with miRNAs is further strengthened as lncRNAs were shown to be characterized as tumor-suppressive or oncogenic and also correlated to drug efficacy as with melittin, a peptide derived from the venom of the honeybee. Melittin was shown to increase PDA sensitivity to gemcitabine via regulation of the cholesterol pathway and concomitantly alters the lncRNA expression profiles in these cells, upregulating lncRNA NONHSAT105177, found within PDA EVs. Upregulation of NONHSAT105177 and transfer via EVs to PDA cells reduced their EMT and migratory potential and suppressed tumor growth. Although performed on an uncommonly used drug in the clinic, this study provides insight into the role of lncRNAs in regulating drug sensitivity and efficacy and should be further investigated in the context of clinically relevant therapies [34].

#### 2.1.3. Circular RNAs

Lastly, circular RNAs (circRNAs) were characterized as non-coding endogenous RNAs originating from alternate splicing and pre-mRNA processing of a large fraction of genes in eukaryotic cells. Described as abundant, stable and located in the cytoplasm or the nucleus, circRNAs are differentially expressed in various cancer cells and act as ceRNA modulating cancer-related signaling pathways [35]. As for the other class of ncRNAs, circRNAs packaged in EVs are being investigated as biomarkers for the early detection and diagnosis of PDA [36]. They were also correlated with PDA cell repopulation and recurrence following irradiation of PDA patients with a specific correlation of hsa_circ_0002130-hsa_miR_4482-3p-NBN interaction network, modulating metabolic process and lysine degradation [37]. A recent study from Ye et al. further revealed that PDA tumor cells-derived EVs transferred specific circRNAs such as Hsa_circ_0000069, favoring malignant transformation as well as proliferation and migration processes through upregulation of STIL [38]. In the highly invasive subset of liver metastases PDA-EVs, circRNA PDE8A was found in superior quantities, and its expression in PDA patient plasma-derived EVs was associated with greater lymphatic invasion and a higher TNM stage. EVs containing circRNA PDE8A released from highly malignant cells and received by low malignancy cells induce the sequestration of miR-338, again lifting the suppression of MACC1 expression and allowing for activation of the cascade of the Met/Erk/Akt pathway, leading to increased invasive capacity. Animal models of PDA, as well as PDA patients, had elevated levels of plasma-derived EVs containing circPDE8A and correlated with tumor progression [39].

Studies on circRNAs in PDA-EVs remain preliminary, despite the high content of these RNAs in EVs and their correlation with poor clinical characteristics. Further studies must be performed in order to dissect the mechanisms by which EVs-circRNAs promote tumor progression, tumor cell activities and abilities, as well as their correlation with specific clinical characteristics such as tumor recurrence.

#### 2.1.4. Proteins

PDA tumor cells-derived EVs also carry internal as well as transmembrane proteins that were associated with mechanistic improvements of tumor cell abilities as well as gain-of-function in biological outcomes of PDA, such as aggressiveness, metastatic potential or therapeutic resistance. In PDA, a specific isoform of CD44, CDD44v6, was identified as a reliable cancer-initiating cell (CIC) marker. In this cellular context, it acts as a transmembrane receptor primarily for hyaluronic acid and other ECM components and actively regulates the Ras-MAPK pathway promoting metastases, ECM remodeling, resistance to cell death and EMT. Interestingly, CD44v6 expression is correlated to Tetraspanin 8 (Tspan8) expression in PDA tumor cells derived-EVs, described in the context of ECM activation and angiogenesis, and is required for EV uptake in non-CIC cells. CD44v6 and Tspan8-carrying PDA tumor cells derived-EVs, when received by CD44v6 depleted non-metastatic cells, led to interactions of both molecules with Tspan8-associated integrins, CD49f and CD104, concomitant activation of mutual downstream pathways and conferred an adhesive, invasive and migratory phenotype to non-CIC [40]. Deep sequencing revealed altered mRNA and miRNA expression profiles of non-CIC following PDA tumor cells derived-EVs treatment, phenotypically translating to Cd44v6 and Tspan8 concerted non-CIC activation, apoptosis resistance, EMT and higher motility. In order to prevent tumor dissemination, RTK activation, as well as EV-binding and uptake, could be potentially targeted via these two cell-fate determining molecules [41]. Additionally, claudin-7, also identified as a biomarker of CICs, was found to be expressed on PDA CIC derived-EVs and impacted PDA non-CIC motility, invasiveness, metastatic dissemination and lymphangiogenesis. These downstream effects were regulated by enhanced integrin signaling via claudin-7-EVs, as well as RTK and GPCR signaling activation in receiving non-CIC cells [42]. Migration and invasive properties are recurrent gain-of-functions for PDA tumor cells following uptake of PDA tumor cells-derived EVs. Indeed, several studies reported that highly metastatic PDA tumor cells-derived EVs are able to transfer metastatic potential to recipient cells, promoting migration and invasion, as shown by Jin et al. through the transfer of EVs containing zinc finger protein 4 [43] or by Wei et al. through the transfer of EVs carrying the receptor tyrosine kinase Eph receptor A2 (EphA2) [44].

Metabolic reprogramming of PDA tumors is a key feature of these cells, forcing them to adapt their energy demand to the restrictive and stress-inducing tumor microenvironment. In this context, the lipidic content of PDA tumor-derived EVs was found to recruit and activate pancreatic cancer stem cells (CSCs). More precisely, Kuc et al. showed the case of ceramide-1-phosphate, a metabolite of ceramide, recognized as a chemoattractant of CSCs with anti-inflammatory and anti-apoptotic roles [45]. Specific metabolic enzymes were also transferred by PDA tumor cells-derived EVs. Protease asparaginyl endopeptidase (AEP), expressed in several carcinomas and previously associated with increased metastases and MMP activity, was shown to be transferred via PDA tumor cells derived-EVs and consequently, in recipient PDA tumor cells, increased migratory and invasive capacity, going along with the finding that AEP is usually found at the invasive front of tumors [46]. Finally, hypoxic PDA cells, with the aid of upregulated HIF-1-alpha, were found to release larger quantities of smaller-sized EVs and promoted their survival even under extremely hypoxic conditions, indicative of yet another communicative aspect of EV structure and cargo between heterogeneous cancer cells [47].

Lastly, the protein content of PDA-cell-derived EVs was also identified as targets in the treatment of PDAC. Expression of an endoplasmic reticulum protein and receptor for oncogenic soluble protein DKK1, cytoskeleton-associated protein 4 (CKAP4), was reported to be associated with poor prognosis in various solid tumors, including PDAC, acting via the PI3K-Akt pathway, and was recently found to be expressed by PDAC patient sera EVs on their surface. Effectively inhibiting CKAP4 using a monoclonal antibody showed reduced tumor formation and improved survival in mice models. The authors propose using an AKT inhibitor in concert with a CKAP4 inhibitor to target tumor cells and their EVs [48].

Overall, accumulating evidence of the implication of various types of RNAs and protein messengers contained within tumor-cell-derived EVs in PDA diagnosis, as well as aggressiveness and tumor progression, uncovers previously unknown tumorigenic mechanisms through tumor–tumor cell crosstalk. Those gradually add to our knowledge on mechanisms of PDA tumor development and evolution, as well as providing potential novel targets to counter this rapid progression.

### 2.2. Resistance to Treatments

PDA remains an exceptionally aggressive malignancy with a high mortality rate, attributable to late diagnoses at the metastatic stage and tumor plasticity leading to limited response rates to various treatments. Herein, this section will delineate the implication of tumor-cell-derived EVs in transferred resistance between tumor cells to commonly used therapies in clinics to treat PDA, such as chemotherapy and radiotherapy.

#### 2.2.1. Chemoresistance

Several mechanisms in PDA govern resistance to chemotherapeutic agents, such as the transport, activation and metabolism of available drugs, as well as the pronounced contribution of tumor microenvironment cells and their physical structure, conferring resistance to chemosensitive tumor cells. Additionally, emerging evidence points towards chemoresistant tumor cells and transformed cells able to transfer biological content through extracellular vesicles to chemosensitive cells, altering their response to treatments by promoting cell survival and proliferation under treatments.

Interestingly, the physical structure and lipid composition of extracellular vesicles were shown to contribute to chemoresistance, specifically in a PDA model using the intrinsically aggressive MiaPaCa-2 cell line. Using synthetic nanoparticles with the same lipid composition as PDA cell-derived exosomes, Beloribi et al. demonstrated that MiaPaCa-2 cells resist gemcitabine’s anti-proliferative effects via SDF-1a secretion induced by lipids of nanoparticles, ultimately activating the Akt survival pathway passing through the CXCR4-SDF-1a axis [49].

Internal cargo, particularly miRNAs, were associated with PDA tumor cells derived EVs-driven chemoresistance. Yang et al. reported that exosomes derived from PDA tumor cells resistant to gemcitabine mediate horizontal transfer of drug-resistance traits to gemcitabine-sensitive PDA tumor cells. The authors identified the molecular origin of such transferred resistance as the presence of miR-210 in EVs. Following EVs uptake, recipient PDA cells activated the mammalian target of rapamycin (mTOR) signaling pathway, which increased gemcitabine resistance [50]. Another previously identified oncogene in various cancers, miR-155, has emerged as a mediator of chemoresistance. Long-term exposure to gemcitabine increases miR-155 expression in PDA tumor cells and EVs secreted by these cells. Once received by neighboring PDA tumor cells, miR-155 protected receiving-PDA cells from gemcitabine-induced cell death by impairing the activation of pro-apoptotic stress-induced p53 target gene TP31INP1 [51]. Patel et al. further demonstrated the action of miR-155 on the metabolism of gemcitabine. Indeed, the transfer of oncogene miR-155 via EVs from chemoresistant-PDA cells downregulates DCK expression in chemosensitive cells, the gene encoding a gemcitabine-metabolizing enzyme, ultimately reducing the active form of the drug. Consequently, high proliferation rates are maintained, while decreased ROS levels inhibit apoptotic pathways, leading to chemoresistance and tumor cell survival [52]. Alternatively, another exosome-derived candidate for chemoresistance transfer was identified, ephrin type-A receptor 2 (EphA2), described above in transfer of metastatic potential via EVs, which is a receptor tyrosine kinase also found commonly upregulated and responsible for therapy resistance in several other cancers such as breast, cervical and melanoma. EVs carrying EphA2 improve RAS-ERK oncogenesis and tumor angiogenesis [53].

Finally, EVs are also recognized as efficient delivery vehicle systems with low toxicity, biocompatibility, and the capacity to target specific tissues. Chemotherapy results in systemic toxicity, and resistance can be acquired due to poor cellular uptake of drugs. A study highlighted the efficacity of gemcitabine-loaded autologous EVs in the treatment of PDAC, increasing concentration of gemcitabine within the tumor, cytotoxicity in receiving tumor cells, and reduced tumor growth and progression [54]. EVs should be considered as potential carriers of therapeutic agents in combination with chemotherapy, such as by delivery of siRNA targeting mutated KRAS, capable of suppressing tumor progression and increasing survival [55].

Overall, chemoresistant tumor-cell derived EVs are capable of rendering neighboring or distant chemosensitive-tumor cells impervious to the anti-proliferative and pro-apoptotic effects of commonly used chemotherapy reagents such as gemcitabine, demonstrating the importance of EVs anew as major communicating hubs and carriers of therapeutic agents between tumor cells and highlighting them as a target to improve the response to therapy and ultimately patient survival.

#### 2.2.2. Radioresistance

In addition to chemotherapeutic protocols, radiotherapy is under investigation to “downstage” pancreatic tumors, delay recurrence and prolong patients’ survival [56]. However, chemoradiation often results in tumor repopulation, where residual tumor cells proliferate following radiation, causing relapse. Recent studies in various cancers have demonstrated that irradiated, dying tumor cells undergo multiple alterations in signaling, continue to secrete an abundance of soluble factors as well as EVs, which are then received by residual cells. These residual cells, also known as tumor-repopulating cells (TRCs), have significant DNA damage yet were found to be responsible for relapse by rapid proliferation and tumor repopulation. As they are the founding basis of the newly reformed tumor, TRCs were found in part to express cancer stem-cell marker ALDH1. Interestingly, Jiang et al. revealed that dying tumor cells secrete exosomes rich in miR-194-5p, received by TRCs, arresting the cell cycle immediately after radiation and allowing for DNA damage responses, thus favoring the survival of TRCs and their consequent proliferation. Normally a tumor suppressor miRNA inhibits cell proliferation and migration, whereas, in this context, miR-194-5p was expressed transiently, allowing for DNA repair. The surviving TRCs undergo a proliferative boost in the presence of dying tumor cells, attributed to dying cell-secreted PGE2. In a clinical prospect, authors also presented aspirin as a booster of radiotherapy as it can suppress repopulation by inhibiting exosome and PGE2 secretion from dying tumor cells [57]. Conversely, Nakaoka et al. reported that exosomes from irradiated PDA tumor cells sensitize neighboring tumor cells to radiation by increasing intracellular ROS and DNA damage levels in recipient cells, highlighting an opposing impact of irradiated tumor cell-EVs on residual cells [58].

In concert with established studies demonstrating a chemoprotective tumor-stroma crosstalk, along with significant tumor spread under treatment, tumor cell-derived EVs-conferred therapy resistance and EVs-promoting relapse adds to our understanding of the multiple mechanisms giving rise to tumor-cell therapy resistance and could lead to the establishment of a novel and promising method of predicting and following patient responses to certain therapies.

## 3. Stromal Cells EVs-Mediated Crosstalk

The majority of the PDA stromal microenvironment consists of tissue components and its building blocks in transformed and/or increased abundances such as cancer-associated fibroblasts (CAFs), immune cells, adipocytes, nerve cells and endothelial cells. This cell mixture forms the cellular environment in which the heterogeneous PDA tumor cells are embedded. All of these cellular components interact with each other as well as with PDA tumor cells influencing, in a pro- or anti-tumoral manner, PDA development, evolution and resistance to treatments, with a consequent impact on patients’ care and survival. The following studies highlight the implication of EVs in stromal-to-tumor cell crosstalk and its consequent impact on PDA development (Table 2).

### 3.1. Cancer-Associated Fibroblasts (CAFs)

CAFs, also known as tumor-associated fibroblasts (TAFs) or mesenchymal stromal cells, represent a highly heterogeneous group of activated fibroblasts originating from various tissues such as resident tissue fibroblasts (the pancreatic stellate cells or PSC), bone-marrow or adipose-tissue derived mesenchymal stem cells (MSC), as well as endothelial and epithelial cells having undergone endothelial-to-mesenchymal transition/epithelial-to-mesenchymal transition. These activated fibroblasts represent the most prominent cellular component of PDAC. Subject to constant modification and communication with tumor cells, CAFs are responsible for the production of the signature desmoplastic reaction consisting of various ECM fibers and components, which may account for 40–80% of stroma within the tumor [59,60]. It was recently highlighted that CAFs, which were classified primarily as pro- or anti-tumoral, are in fact a mixture of many subtypes clustered depending on their expression of cell marker expressions such as α-SMA, PDGFR-β, fibroblast-associated protein (FAP) and podoplanin, among others or functional properties (inflammatory, antigen-presenting) and proportions of which vary within and between PDA tumors. Keeping this in consideration, we understand better that even with promising preliminary data [61], why global targeting of CAF was and remains unsuccessful. Heterogenous subpopulations with varying marker expression render difficult therapeutic targeting or depletion of CAFs, without contradictory outcomes. Globally, even though not exclusively nor specifically, CAFs have a spindle-shape morphology with abundant cytoplasmic extensions, proliferate actively, migrate and contribute to the desmoplastic reaction by producing ECM components along with cytokine and EVs production, all serving as signaling hubs. Compared to their quiescent counterparts, such features characterize transformed fibroblasts, those found during inflammation, tissue repair and regeneration, coining the term “a wound that never heals” to the tumor microenvironment, since these cells constantly remain active and modulated through exchanges with PDA cells and contribute to immune invasion, tumor cell migration, metabolic shifts and angiogenesis.

#### 3.1.1. Normal Fibroblast Activation and CAFs Reprogramming

Reprogramming of fibroblasts to pro-tumoral CAFs, demonstrated by increased proliferation, migration, secretory activity and expression of certain markers, may occur through a combination of tumor-secreted EVs and soluble factors, such as IL-6 [62,63]. Tumor cell-derived EVs from various solid tumor types, containing TGFb1 tethered by surface betaglycan, cause the emergence of a myofibroblast phenotype with a rearrangement of the cytoskeleton to a-SMA-abundant and also increases FGF2 secretion, associated with stromal pro-tumoral activity, likely caused by TGFB1 and other factors found in EVs [64]. This led to the investigation of a similar mechanism in PSCs, quiescent fibroblasts residing in peri-acinar spaces that remain so until tissue injury or neoplasia occurs in surrounding ducts. PDA-EVs were found to be directly and solely capable of transforming primary PSCs into CAF-like, potentially attributed to a miRNA, miR-1260, found in abundance in PCC-EVs and acting indirectly on the expression of TGFb and TNF in PSCs [65]. Along with an increased PDGFRb expression as in CAFs, another study demonstrated that PSCs are preferentially trafficked to metastatic sites under the influence of PDA-EVs, via activation of the PDGFB pathway in distant cancer cells, hypothesizing that PDGFB could act as a chemokine to attract PSCs. However, tumor cells were required to already be present at the future metastatic site in order for EVs to be able to enhance PSC migration and recruitment, as EVs are not a source of PDGFb [66]. Besides miRNAs and TGFb, Novizio et al. reported that Annexin A1 released in PCC-EVs drives a mesenchymal switch and turns fibroblasts into myofibroblasts through Formyl Peptide Receptors (FPRs) activation [67]. Tumor cell-derived EVs isolated from PDA patient sera, as with other carcinoma cell lines, mediate the transfer of mRNA, such as that of telomerase—hTERT mRNA—to non-telomerase expressing somatic cells, fibroblasts, marking them with augmented proliferation and delayed senescence, as well as protection from DNA damage and subsequent apoptosis. It is hypothesized that in an in vivo setting, constant exposure of fibroblasts to tumor-EVs and hTERT mRNA transfer can significantly increase their lifespan and might even make them a group of non-malignant immortalized cells within the tumor microenvironment, constantly present to serve the tumor [68].

Finally, besides CAFs heterogeneity and activation, the impact of PDA tumor-derived EVs on fibroblasts may also influence protein post-translational modifications and endosomal trafficking pathways to ultimately influence CAF functions. Specifically, pancreatic cancer cells with p53 gain-of-function mutations are known for their enhanced migratory capacity, compared to p53 null genotypes, by promoting recycling through endocytic pathways of integrins and EGFR1, dependent on the presence of the Rab11 effector, Rab-coupling protein (RCP) [69]. Mutant p53, with the influence of Rab35, restrain the secretion of podocalyxin (PODXL) in EVs, and thus promote increased alpha-5-beta-1 integrin recycling in CAFs along with increased migration and the deposition of more branched, disorganized ECM fibers in both the pancreas and the lung, a primary site of PDA tumor metastases. The reseeding of tumor cells on this modified ECM led to increased migration, suggesting that it could ultimately influence tumor cell spreading at the primary site, as well as extravasation, colonization and dormancy at the metastatic site [70]. Thus, the genotype of tumor cells plays a role in these processes through a modification of EV content and ECM architecture to ultimately promote invasion and metastases formation, using fibroblasts as the messenger. Altogether, these studies demonstrate that PDA tumor cells-EVs drive CAFs activation and modify their abilities to serve tumor cells.

#### 3.1.2. Fibroblasts/PSC/CAFs-Derived EVs Impacts on PDA Tumor Cells

This variety of fibroblast, from a normal state to a broad panel of fully activated CAFs, explains the consequent, multiple impacts of this cellular compartment on PDA tumor cells. While normal fibroblast-EVs, carrying miR-520b to tumor cells, were determined to be tumor suppressive and highlighted their potential therapeutic role [71], EVs derived from either PSC or CAFs were reported as pro-tumoral. In fact, PSC-derived exosomes were found to contain upregulated levels of miRNAs compared to their cell counterparts, such as miR-21-5p and miR-451a, and when received by PDA tumor cells, induced their proliferation, migration and chemokine expression [72]. PSC-derived exosomes, containing miR-5703, were further demonstrated as promoting PDA tumor cells growth through a miR-5703 mediated downregulation of CMTM4 and the consequent activation of PI3K/Akt pathway [73], while miR-21 was shown to promote PDA tumor cell migration by enhancing Ras/ERK pathway activity [74]. Finally, under hypoxic conditions, PSC-derived EVs were reported to promote proliferation and invasion of PDA tumor cells through the transfer of miR-4465 and miR-616-3p, which then target PTEN and activate AKT signaling in PDA tumor cells [75].

Similar to PSCs, CAFs-derived EVs were reported as strong tumor-promoting actors playing at various levels such as aggressiveness potential, metabolism and chemoresistance of PDA tumor cells. For instance, our group demonstrated that primary CAF co-cultivated with macrophages in hypoxic and lipid-deprived medium produced ANXA6+ EVs, which, once uptaken by cancer cells, increased their aggressiveness through enhanced survival and migration in vitro. We have also shown that these stromal-derived EVs increased, in vivo, the number of metastasis and were correlated with PDA aggressiveness in human PDA cohorts [76]. The impact of CAFs-derived EVs was also described as being, in part, responsible for PDA tumor cell survival in the hostile, nutrient-deprived and hypoxic environment of PDA. Patient CAF-derived EVs, received via macropinocytosis by PDA tumor cells, block mitochondrial oxidative phosphorylation by inhibiting the electron transport chain and in place provide a plethora of intermediate metabolites, lipids and amino acids, allowing cancer cells to favor glycolysis, reductive glutamine metabolism and enhanced proliferation [77]. Additionally, studies reported the pro-chemoresistance impact of CAFs-derived EVs on PDA tumor cells. Indeed, it was found that CAFs are inherently resistant to gemcitabine, a common chemotherapy regimen for PDA, and when exposed to this drug, they increased EVs release, carrying Snail1 and its downstream target miRNA146a. Such drug-resistant EVs rendered PDA tumor cells resistant to gemcitabine, evidenced by their increased survival and proliferation. Subsequent inhibition of EVs biogenesis by CAFs via a drug, GW4869, reduced survival in CAF-cocultured PDA cells, highlighting the potential of targeting EVs as a therapeutic option [78]. Similar results were obtained following the transfer of CAF-derived EVs carrying miR-106b to PDA tumor cells [79]. While these studies demonstrate the impact of fibroblast-derived EVs on PDA tumor cells, contributing to several hallmarks of cancer, they raise all the more important questions on the potential broader impact of fibroblast-derived EVs on PDA tumor cell biology and capacities, and thus their consequent role on PDA development and patients’ fate.

### 3.2. Endothelial Cells

One of the key features of PDA is the lack of oxygen supply, creating a hypoxic environment due to scarce vascularization. However, for tumors to grow past a 2 mm^3^ diameter, invade, circulate systemically and seed in metastatic organs, they utilize the process of angiogenesis, normally essential for developmental processes and recovery following tissue injury, forming new blood vessels from a pre-existing vascular network. This process, strictly and chemically regulated by a balance of activators and inhibitors, requires the degradation of the basement membrane of a vessel, endothelial cell (EC) proliferation, migration, sprouting and formation of new branches [80]. Known activators include growth factors, mainly vascular endothelial growth factor (VEGF) upregulated via hypoxia-inducible factor-1 alpha (HIF-1a), FGF, angiogenin, TGF, hepatocyte growth factor (HGF), cytokines interleukins-1, -6, -8 and matrix metalloproteases (MMPs) amongst others, whereas some inhibitors include interleukins-10, -12, tissue inhibitor metalloproteases (TIMPs) and angiogenin [81]. Since EVs carry such bioactive molecules and diffuse systemically in the organism, interest in communication between PDA tumor cells and endothelial cells via EVs to promote primary tumor growth, invasion, and metastases has gained significant interest.

#### 3.2.1. Activation and Angiogenesis

An early study of tumor-EV content involved in angiogenesis demonstrated the abundant presence of cell membrane and EV-bound tetraspanin-8 (Tspan8), upregulated VEGFR and CD31 in endothelial cells (ECs), accelerating angiogenesis in a rat model of PDA [82]. Additionally, they identified an angiogenic loop, as tetraspanin-8 is upregulated in newly sprouting vessels. It was hypothesized that T-span8 interacts with integrins for MMP transcription or with G-coupled protein receptors (GPCRs), regulating various signaling pathways. In subsequent studies, overexpression of Tspan8 was found to selectively recruit CD49d into EVs, which is essential for their uptake and EC activation in a VEGF-independent manner, specifically promoting proliferation and migration as well as survival and differentiation of EC progenitors [83,84]. Almost a decade later, more simplified in vitro incubation of pancreatic tumor cell-derived EVs with human umbilical vein endothelial cells (HUVEC) led to observations of dynamin-dependent endocytosis of tumor-EVs and enhanced tube formation associated with the activation of Akt and ERK1/2 in ECs [85]. Similarly, the lncRNA UCA1 and CCAT1, contained in PDA tumor EVs, were shown to promote angiogenesis in hypoxic conditions through miR-96-5p regulation [86] and miR-138-5p/HMGA1 axis [87], respectively.

#### 3.2.2. Extravasation and Metastasis

Apart from their involvement in the production of new vessels serving the tumor, endothelial cell activity dictates extravasation and intravasation of tumor cells from the primary tumor to vessels of a metastatic niche. Functional endothelial cells restrict vessel permeability via tight junctions and the basal lamina, forming a sealed barrier and selective permeability of molecules passing through or between cells of the monolayer. Vessels of the tumor microenvironment are often malformed and leaky, formed by rapid and uncontrolled angiogenesis, bypassing the criteria of barrier integrity. Tumors are capable of remodeling vessels in order to serve themselves better, mainly through the action of VEGF on the Src/VE-Cadherin axis, leading to internalization of cadherin complexes and loss of adherens junctions, thus increasing vessel permeability [88]. Predictably, tumor-derived EVs are not exempt from carrying regulators of such bioactive molecules as was found in a study of circular non-coding RNA, circ-IARS, isolated from PDA primary and liver metastatic tumor cells and correlated with poor postoperative survival time and high metastatic burden. Circ-IARS acts as a sequester of tumor suppressor miR-122 and, via the RhoA pathway, is able to increase F-actin expression and cell contractility as well as a loss of tight junction protein zona-occludens-1 (ZO-1) in HUVECs, enhancing endothelial cell permeability and allowing for passage of tumor cells between cells [89]. In other cancers such as hepatocellular, colorectal and breast cancer, exosomal miRNAs such as miR-25-3p, miR-103, miR23a and miR939 had a similar effect of reducing vessel integrity via their actions on VE-cadherin complexes and ZO-1 localization and gene expression.

#### 3.2.3. Coagulation Regulation and Thrombosis

The highly inflammatory and pro-coagulant systemic impact of pancreatic tumor secretions, the physical burden on blood vessels of the tumor mass, as well as side effects of treatments and surgery is coupled with hypercoagulability, predisposing patients to venous thromboembolism (VTE), with an almost 60-fold increase in relative risk especially in late-stage disease [90,91]. The coinciding systemic circulation of tumor cells, tumor-EVs and thrombotic factors warranted the study of communication between such elements with patient survival and therapy efficacy. It was found that pancreatic cancer patients have significantly higher levels of circulating microparticles (MPs), loaded with tissue factor (TF), responsible for the activation of the coagulation cascade and platelet aggregation. TF expression in PDA is associated with higher VEGF expression, microvessel density and development of VTE [92]. Linking these two phenomena, a study uncovered augmented thrombosis formation following chemical or laser-induced injury in a mouse model with PDA-EVs containing active TF and P-selectin glycoprotein ligand 1 (PSGL-1) essential for fibrin generation in thrombi [93]. This outcome was unique to and indispensable by cancer-derived MPs and not cancer cells alone. PSGL-1 in MPs accumulated specifically at the site of injury and TF, along with the negatively charged phospholipids on their surface, leading to the generation of thrombin and fibrin. The presence of minimal amounts of tumor-MPs also reduced bleeding and clot formation time. Along with cancer-derived MPs, platelet and endothelial cell-derived MPs were also found in abundance in the presence of PDA tumors; however, a mechanism of inducing coagulation through the activity of all three types of MPs and their cross-talk remains unknown [94]. Furthermore, BxPC3-derived MPs with TF and procoagulant phospholipid phosphatidylserine initiated and amplified thrombin in both cancer cells and cell-free plasma [95]. It was also established that PDA-derived MPs co-express TF pathway inhibitors and integrins a5b1 and a5b3 and actively interact with platelets, promoting tumor growth and metastases formation [96]. Additionally, a biomarker study identified the last aspect of the fibrogenic pathway, fibrin, to be present on circulating MPs and in great abundance in pancreatic and colorectal cancer patients [97].

Pro-coagulatory and pro-thrombogenic EVs interact simultaneously with endothelial cells, altering hemostatic balance and impacting metastases formation. When HUVECs are exposed to TF-positive PDA-EVs, they upregulate levels of E-selectin, a pro-adhesive molecule capable of arresting tumor and myeloid cells, and secrete IL-8, a pro-inflammatory cytokine responsible for inflammatory cell recruitment and chemokine secretion in metastases [98]. Thrombin generation by HUVECs is increased upon exposure to PDA-EVs with TF, and a direct correlation between TF quantity on EVs and subsequently in ECs was established, indicating a transfer of TF from EVs. Interestingly, this shift to a procoagulant phenotype in ECs was maintained in daughter generations, pointing to a potential genetic or epigenetic influence of EVs on ECs [99].

#### 3.2.4. Therapeutic Targets

The analyses of EV contents have led to the identification of potential anti-angiogenic targets. Annexin-A1 (ANXA1) could be one of these targets as ANXA1-containing EVs are found to be elevated in the serum of various cancer patients, and ANXA1 was heavily reported to be involved in PC invasion and metastases processes. ANXA-1-containing EVs enhanced endothelial cell migration and proliferation and tube formation compared to EVs lacking ANXA-1, where ANXA1 is also actively involved in both inward and outward vesicle trafficking [100]. Similarly, myoferlin in PDA-EVs was investigated, being a membrane protein involved in plasma membrane repair and fusion and cell motility, associated with poor prognosis and shown to be implicated in raising VEGFA levels and functional blood vessels in the TME [101]. Unsurprisingly, myoferlin-containing EVs had the same effect on HUVECs with increased migration and proliferation. Myoferlin is also required for EVs uptake, and its presence alters the content of EVs, such as iron metabolism proteins, exosome marker and pro-tumoral protein CD63 and RAB7A, respectively, revealing the merging of multiple EVs effectors and their pathways [102]. Moreover, PC-derived EVs containing miR-27, identified as an oncogene in many cancers, cocultured with human microvascular endothelial cells (HMVECs) led to the upregulation of VEGF and increased proliferation and microvessel density through its action on downregulating the expression of a tumor suppressor, B-cell translocation gene-2 (BTG2) [103,104]. Interestingly, a combination of three miRNAs, miR23A, miR21 and miR 27A can synergetically induce cell proliferation, necessitating an in-depth analysis of implicated signaling pathways and combined inhibition of vesicular miRNAs [105].

In the stressful nutrient-deprived and hypoxic tumor microenvironment, tumor cell-derived EVs have now been established as key players in the promotion of angiogenesis, augmented endothelial cell and vessel permeability, and comorbidities such as hypercoagulability, via a multitude of key signaling messengers. Tumor cell-derived EVs create a constant communicative loop between systemically circulating platelets and endothelial cells, primarily promoting metastases and thrombin formation, necessitating an in-depth investigation of overlapping activated pathways via EVs and their subsequent blockade to halt these processes.

### 3.3. Adipocytes and Nerve Cells

While CAFs, endothelial cells and immune cells (Section 3.4) constitute the main cellular components of the PDA intratumoral microenvironment, other cellular partners, such as adipocytes and nerve cells, are also part of this complex cellular network. To date, few studies depict how extracellular vesicles are implicated in the cellular communication involving adipocytes and nerve cells. Intriguingly, PDA-derived EVs were shown to induce lipolysis in adipocytes of PDA patients through the activation of p38 and ERK1/2 MAPKs and the phosphorylation of hormone-sensitive lipase [106]. This process was further studied and validated in human samples as well as in mouse models of PDA, and authors provided new insights into the time course of metabolic and soft tissue changes in pre-diagnostic PDA and provided preliminary evidence for an EVs-based potential early novel biomarker of PDA [107]. The numerous evidence linking adipocytes-derived EVs to diabetes mellitus, a major risk factor of PDA, further confirm the importance of deepening the uncovered EVs-mediated connection between adipocytes and PDA. Finally, while no studies reported the implication of EVs in the crosstalk between PDA tumor cells and nerve cells (neurons or Schwann cells), it seemed crucial to mention how important this connection might be. Indeed, this EVs-based emerging crosstalk between tumor cells and intra-tumoral nerve was already reported in several solid tumors [108], and nerves are well-known contributors of pancreatic carcinogenesis and metastatic spreading, with neural/tumor communications established revealing a strong neuro-tropism to PDA [109]. Such evidence suggests an influence of EVs-driven crosstalk between tumor cells and nerves into PDA. This concept needs to be deeply investigated as it may have a crucial impact on PDA patients’ care by developing therapeutic tools targeting PDA-associated neuropathies and perineural invasion.

### 3.4. Immune Cells EVs-Mediated Crosstalk

The immune landscape of the PDA microenvironment, initiating in early carcinogenic stages and evolving with disease progression [110], is mainly described as immunosuppressive, including M2 macrophages, T-regulatory (Treg) lymphocytes and myeloid-derived suppressor cells (MDSCs), which outnumber CD8+ cytotoxic T cells, dendritic cells (DCs), natural killer (NK) cells and M1 macrophages. Although tumor-derived immunomodulatory soluble factors are known to alter immune cells towards a more immunosuppressive phenotype, the role of tumor-derived EVs in the evasion of anti-tumor immune responses is being investigated. For instance, it was found that metastatic melanoma cells secrete PD-L1-expressing EVs, responsible for systemic T-cell suppression via PD-1 binding and facilitate tumor growth [111]. Expanding our understanding of EVs-mediated crosstalk between PDA tumor cells and immune cells could highlight mechanisms of immunotherapy resistance in pancreatic ductal adenocarcinoma [112], leading to new strategies to directly augment T cell responses and enable an immune-mediated control of this malignancy.

#### 3.4.1. Monocytes

An essential component of the innate immune system and a regulator of the adaptive one, monocytes are a set of heterogeneous mononuclear phagocytic cells originating in the bone marrow then freely circulating in the blood to finally localize into tissues to give rise, in tissue and context-dependent manner, to dendritic cells (DCs) or macrophages. Recent studies have shown that monocytes consist of different subsets (classical, intermediate and non-classical) according to a panel of cell-surface markers (as CD14 and CD16): activation of transcription factors and distinct phenotypical and functional features. To date, studies reported the classical monocytes as responsible for homing to a site of inflammation and the subsequent recruitment of other immune cells while non-classical ones patrol the vasculature for damages to the endothelial wall and extravasate during inflammatory conditions. Monocytes were vastly investigated in the context of neoplasia as they are capable of influencing not only cancer immunosurveillance but also the desmoplastic reaction, angiogenesis and metastases. From a pro-tumoral standpoint, monocytes were found to give rise to tumor-associated macrophages, recruit Treg cells, inhibit cytotoxic T cell activity, promote angiogenesis and model the extracellular matrix. On the other hand, monocytes harbor anti-tumoral functions such as direct cancer cell cytotoxicity and engulfment, inhibition of Treg lymphocytes and cooperation with NK cells.

Conflicting studies on their global influence on tumor progression merit further studies to explain such disparate heterogeneity better considering other aspects of the tumor microenvironment, varying stimuli and intercellular communication via EVs. Recently, it was confirmed that monocytes are capable of interacting with and engulfing tumor material, including tumor-EVs, both from primary tumors and circulating tumor cells in the vasculature of the lung, a primary site of metastases for many cancers [113]. In PDA, tumor-derived microvesicles (TMVs) were reported to transfer several surface determinants and mRNA from tumor cells to monocytes [114]. A subset of TMVs, CD44vH^+^, were found to be integrated into monocytes via an alternatively spliced version of CD44, CD44H, leading to decreased secretion of inflammatory chemokines CCL4 and CCL5 in a STAT3-dependent manner [115]. TMVs are capable of transferring and inducing chemokine production in monocytes as well as promoting angiogenesis in a mouse model. Classical monocytes exposed to TMVs containing hyaluronan had increased expression of anti-inflammatory and immunosuppressive cytokine IL-10, mediated by the PI3K/Akt/mTOR pathway, making the extracellular matrix a potential mediator of the interaction between tumor-derived vesicles and immune cells [116]. In the tissue context, monocyte survival is limited, leaving cells to differentiate or undergo apoptosis. While for several solid tumors, the monocytic pro-survival role of tumor cells EVs was demonstrated, this potent role of PDA tumor cells-derived EVs remains to be uncovered, and how it may contribute to the immunosuppressive landscape of the PDA tumor microenvironment.

In order to better delineate the role of peripheral immunity in tumorigenesis as opposed to the localized immunosuppressive environment in patient tissues, a distinct monocytic immunophenotype in pancreatic cancer patients was identified. Indeed, following priming by PDA tumor cells exosomes, a higher quantity of an immunosuppressive monocytic subset, CD14^+^/HLA-DR^lo/neg^, in peripheral blood was highlighted in a group of patients, correlating with repressed T-cell activity and shorter survival [117]. Tumor-derived EVs can be a source of activating cytokines, regulatory miRNAs and signaling molecules, all capable of modifying the phenotype of monocytes. Interestingly, the recruitment of bone-marrow monocyte populations to the tumor via blood-circulating tumor-derived EVs remains an uncovered subject.

#### 3.4.2. Macrophages

Although monocytes are just now emerging as an important precursor of the immunosuppressive tumor microenvironment, tumor-associated macrophages (TAMs) have been a topic of intense scrutiny over the recent decades. Macrophages are leukocytes with the highest infiltration in PDA and were found to promote tumor cell invasion and chemoresistance. Thus far, macrophages were classified in polarized states; M1 being the anti-tumoral and classically activated macrophages, and M2, the pro-tumoral type, TAMs, that correlate with poor prognosis in many solid tumors as in PDA [118], and in between M1 and M2 macrophages there is a large variety of intermediate states. TAMs facilitate tumor growth and metastases by secreting various enzymes and cytokines associated with ECM remodeling.

##### Macrophages Polarization

Using a pancreatic cancer cell line, AsPC-1 (ascites-derived metastatic cells), it was shown that tumor cells derived-EVs are capable of inducing the expression of M2 macrophage markers, such as CD163 and CD206, and the secretion of cytokines and pro-angiogenic factors. These exosomes also contained high levels of bioactive arachidonic acid (AA), allowing for an increased fusion rate with macrophages [119]. Such impact of PDA tumor cells derived EVs on macrophage polarization was confirmed by a study indicating that pancreatic cancer cells generate miR-301a-3p-rich exosomes in a hypoxic microenvironment, which then polarize macrophages towards M2 phenotype to promote malignant behaviors of pancreatic cancer cells [120]. The presence of ezrin in PDA tumor cells-derived EVs was also associated with M2 macrophage polarization and metastasis promotion [121]. Interestingly, it was reported that polarized states of macrophages could be regulated via microRNAs-loaded EVs. Using targeted nanoparticles, miR-155 and miR-125b, overexpression in PDA cells reversed previously polarized M2-polarized macrophages to M1, evidenced by increased expression of IL-1b and iNOS [122]. Thus, PDA tumor cells-derived EVs are capable of directly reprogramming TAMs found in the tumor microenvironment through the horizontal transfer of EVs carrying regulatory factors.

In line with the capacity of EVs to reprogram macrophages, it was demonstrated that artificially modifying the content of PDA-cell-derived EVs allows repolarizing previously tumor-cell polarized M2 macrophages into M1 macrophages. In one study, authors used transfection of the pancreatic cancer cell line, PANC-1, with miR_155 and miR-125B using a specialized hyaluronic-acid nanoparticle delivery system in order to modify EVs’ contents secreted from these cells. Modified EVs carrying these miRNAs were capable of re-polarizing M2 macrophages back to M1 macrophages [122]. In another study, it was demonstrated that bone marrow mesenchymal cell-derived EVs loaded via electroporation with oxaliplatin and siRNA targeting galectin-9, which by binding dectin-1 on macrophages leads to the formation of TAMs, are capable of causing tumor cell apoptosis and immunogenic cell death via oxaliplatin and interestingly reprogram M2 macrophages into M1 macrophages, all the while targeting PDA tumors specifically and remaining active in circulation [123]. These studies effectively highlight the robust impact of intercellular communication via EVs in a therapeutic context and show the importance of further investigating the potential of EVs as stable, non-toxic and deeply tissue-penetrating carriers of therapies.

##### Macrophages-Derived EVs

Conversely, several studies reported the impact of macrophages-derived EVs, mainly M2, on PDA tumor cells’ aggressiveness and their augmented oncogenic abilities. Yin et al. reported that M2 macrophages-derived EVs deliver lncRNA SBF2-AS1 to PDA tumor cells, upregulating the X-linked inhibitor of apoptosis protein (XIAP) axis, to promote PDA tumor cells survival [124]. Apart from pro-survival effects, M2 macrophage-derived exosomes were shown to promote PDA cell migration and invasion by transferring miR-501-3p and thus targeting TGFβR3 in PDA tumor cells [125]. A recent report highlighted that M2 macrophage-derived EVs, carrying miR-21a-5p, stimulate the differentiation and activity of PDA stem cells through targeting KLF3 [126], a transcription factor known to act as a tumor suppressor gene in cancers by suppressing stemness-related genes [127]. Finally, macrophages-derived EVs can also impact PDA patients’ fate by conferring chemoresistance to PDA tumor cells. This process was highlighted through the transfer of miR-365 impairing gemcitabine activity [128]. Such chemoresistance transferred by macrophages-derived EVs was also observed by Xavier et al., who reported that chitinase 3-like-1 and fibronectin, in the cargo of extracellular vesicles shed by human macrophages, influence pancreatic cancer cellular response to gemcitabine [129].

The constant bidirectional communication between macrophages of the TME and tumor cells via EVs highlights the grave importance of intercellular communication in PDA evolution, as tumor cell-derived EVs drive macrophages to an anti-inflammatory, tumor-growth and metastases promoting state, and consequently, these pro-tumoral macrophages directly impact tumor cell abilities and response to therapeutic reagents.

#### 3.4.3. Dendritic Cells (DCs)

PDA tumors also recruit the primary antigen-presenting cells (APCs), dendritic cells (DCs), as part of their arsenal. As with monocytes, DCs originate in the bone marrow from a separate bipotent progenitor, leading to the development of lymphoid- and non-lymphoid tissue-resident DCs (classical DCs-1 and -2) or circulating plasmacytoid DCs (pDCs). Monocytes give rise to context-dependent inflammatory DCs within tissues and tumors. When mature, DCs act by presenting antigen-bound MHC-II molecules to T-cells, along with costimulatory receptors, to subsequently activate CD4^+^ and CD8^+^ T cells as opposed to their immature state, which facilitates immune tolerance. In the context of cancer, although the inflammatory environment allows for the recruitment of DCs, they are mostly defective in activating an anti-tumor response supposed to activate cytotoxic T-cells due to immune escape mechanisms, such as suppression of immune checkpoints and expansion of immature DCs, among others. Using a mathematical model to decipher PDA tumor cells and DCs communication, it was reported that pancreatic cancer cells shed exosomes that inhibit immune responses by DCs, while DCs secrete exosomes that induce apoptosis of pancreatic cancer cells [130].

##### Dendritic Cells Maturation

Tumors can actively give rise to an immunosuppressive DC population, the mechanisms of which remain poorly understood. However, the mechanism of DCs modification to facilitate immune escape via tumor cells-derived EVs is just now beginning to be analyzed. PDA-derived EVs are capable of downregulating the expression of DAMP/PAMP receptors TLR4 on DCs, via miR-203, leading to decreased secretion of DC maturation cytokine TNFα as well as T-cell activators IFNy and IL-12 [131]. A comprehensive analysis of PDA tumor cells-derived EVs miRNA and EVs-treated DC mRNA expression profiles led to the identification of the transcription factor of MHC-II, regulatory factor X-associated protein (RFXAP), inhibited in PDA by EV-derived miR-212-3p. Low levels of MHC-II are associated with poor prognosis and histological grade, potentially due to dysfunctionalities in the RFX protein complex containing RFXAP, promoting tumor immune tolerance and invasion [132]. Additionally, DCs treated with PDA tumor cells-derived EVs had varied lncRNA and mRNA expression profiles, with downregulation of genes potentially leading to DC-mediated immune escape such as lgmn, an enzyme involved in the processing of toxins and endogenous proteins for MHC-II presentation [133].

##### Dendritic Cells-Based Therapies

In the goal of enhancing DC-based immunotherapy by using tumor cells-derived EVs as immune agonists of DC activation, authors depleted tumor-derived exosomes of all miRNAs and demonstrated that T-cells activated by DCs receiving these exosomes had higher perforin and cytotoxic secretions [134]. Although effective in enhancing the overall anti-tumor activity, in vivo studies would be required to attest to the efficacy of exosomes as immune agonists and of such miRNA-elimination techniques in organisms. In a PDA model, a similar efficacy of vaccination with autologous DCs activated by PDA tumor cells-derived EVs presentation was found. Tumor cell-derived EVs carry a multitude of tumor antigens, are readily uptaken by DCs via high expression of exosome ligand, LFA1, facilitated by CD81, and are preferentially loaded into the MHC-II complex. However, the overbearing presence of myeloid-derived suppressor cells (MDSCs), recruited by the PDA stroma, reduces T-cell infiltration and activation and surpasses recruitment of PDA tumor cells-derived EVs loaded DCs (TEDCs), hampering the efficacy of vaccination. Such data warrant the combination of TEDCs with chemotherapeutic drugs blocking MDSC maturation, pushing their differentiation or apoptosis, ultimately prolonging survival and reducing metastases formation. Combining drugs such as all-transretinoic acid (ATRA), a tyrosine-kinase inhibitor Sunitinib (Sun), and a commonly used chemotherapeutic reagent gemcitabine (GEM) with TEDCs effectively activated the immune system in mice and reduced stromal reaction with disorganization of α-SMA^+^ cells [135]. However, it should be considered that TEDCs were not specifically homed to the tumor without several injections and repeated applications of this cocktail led to an immune system collapse in mice, calling for additional modifications to the existing protocol and analysis of patient-specific MDSC infiltration to determine valid clinical procedures. Targeting the impact of tumor cell-derived EVs on dendritic cells could be a promising avenue to improve the maturation of antigen-presenting cells and the infiltration of T-cells.

#### 3.4.4. Myeloïd-Derived Suppressor Cells (MDSCs)

The relevance of tumor genotype, as with CAF and ECM production, and sustenance of an immunosuppressive microenvironment induced via tumor cells derived-EVs was also investigated with a specific focus on SMAD4 loss, associated with worse prognosis and increased cell proliferation and metastases, a unique characteristic of gastrointestinal cancers and representative of upwards of 50% of PDA profiles. PDA tumor cells-derived EVs contribute to MDSC expansion, particularly monocytic MDSCs (mMDSCs)—with higher immunosuppressive activity than granulocytic or neutrophil-derived MDSCs, (gMDSCs). Interestingly, this effect was only observed for SMAD4^+^ PDA tumor cells-derived EVs. EVs content from SMAD4^+^ and not SMAD4^−^ PDA cells revealed upregulation of hsa-miR-1260a, leading to the accumulation of calcium and expansion of MDSCs. Furthermore, SMAD4 PDA tumor cells-derived EVs overexpressed glycolytic enzymes and subsequently transferred these to myeloid cells, increasing glucose consumption and lactate production, highlighting the Warburg effect in immune cells [136]. Overall, SMAD4 loss is associated with a more aggressive phenotype, attributed to tumor cells derived-EV transfer to myeloid cells, increasing their expansion and immunosuppressive activity, and favoring a pro-tumoral environment.

#### 3.4.5. Mast Cells (MCs) and B lymphocytes

Other less frequent populations of immune cells are concurrently investigated, including mast cells (MCs), another heterogeneous immune population largely involved in IgE-activated allergic inflammatory reactions. In cancer, they are known to have either pro-, through the secretion of angiogenic factors, or anti-tumorigenic effects, by initiating an immune response, depending on the type of cancer and their localization with respect to the tumor. To date, only one study has highlighted the effects of PDA tumor cells derived-EVs on MCs, resulting in their activation via EV-cell membrane receptor-mediated activation of ERK-1,2 signaling, leading to the upregulation of tissue-remodeling genes and the secretion of IL-6, IL-8, and VEGF [137]. However, the after-effects of MC activation in tumor progression and therapy resistance are largely unknown. The humoral immune response in cancer is also an area of interest, with limited knowledge of its effects in facilitating an anti-tumor response via tumor-derived EVs. B lymphocytes are responsible for the production and secretion of autoantibodies targeting tumor-associated antigens (TAA), which appear preceding neoplasia, not based on protein mutations but the localization and post-translational modifications of such proteins within tumor cells. Tumor cell-derived EVs are a reservoir for TAAs, highly represented on the surface membrane of EVs and are targets of freely circulating anti-TAA autoantibodies in the plasma of PDA patients. As with TEDCs, many studies have pointed to the efficacy of inducing an immune response with exosome-bound TAAs as opposed to the soluble form in order to activate T lymphocytes and allow the production of autoantibodies effectively. Tumor cell derived-EVs sequester circulating autoantibodies via their strong expression of TAAs and thus inhibit complement-mediated and autoantibody-based cytotoxicity [138]. These PDA patient autoantibodies are more specifically reactive to tumor exosomes than normal ones, emphasizing the role of EVs as bait for humoral immunity and ultimately protecting tumor cells from death.

#### 3.4.6. Natural Killer (NK) Cells

The resistance to such humoral immunotherapies arises due to the requirement for constant recognition of tumor antigens and production of autologous antibodies, a step which can be bypassed by the rapid cytotoxic activity of innate immune cells, natural killer (NK) cells, mediated by direct cell–cell contact with their targets through coordinated stimulation of activating and inhibiting receptors [139]. In comparison to blood cancers, solid tumors, especially PDA, do not have sufficient proportions of activated natural killer cells, with less than 0.5% of NK cells in comparison to up to 10% for other solid tumors such as breast, lung, stomach and colorectal, attributable to the lack of CXCR2 receptor required for their homing. Additionally, NK cells present in PDA do not co-express activating receptors, NKG2D and DNAM-1, limiting their survival and proliferation in the extremely hostile and hypoxic PDA tumor microenvironment. Ex vivo expansion of NK cells in PDA patients led to prolonged disease-free and overall survival, necessitating the increasing attention given to these innate immune populations [140]. Interestingly, NK-derived EVs, enriched in miR-3607-3p, were shown to inhibit the malignant transformation of PDA tumor cells through directly targeting IL-26 [141].

In PDA, NK-cells positive for its activating receptors, such as NKp30 and NKp46, are found in reduced quantities, leading to cellular dysfunction and low tumor cell killing [142]. It was speculated that tumor cell derived-EVs, as well as soluble BCL-2-associated athanogene-6 (BAG6), can block NKp30, paralleled to infection with certain viruses, raising the question on the mechanism of NK cell dysfunction. Exposure of NK cells to PDA tumor cells derived-EVs led to a decrease in NKG2D receptors expression and of CD107, a functional marker of NK cells, as well as a reduction in the secretion of IFNγ and TNFα, two essential cytokines for NK cell cytotoxicity. A mass spectrometry analysis revealed the presence of immunomodulatory molecules in these EVs, including TGFb1, nectin-2 and PVR, the former identified as responsible for the downregulation of NK-activating receptors, reduced glucose uptake and decreased cytokine production in PDA, as well as hypoxic lung and leukemia cancer models [143], and found in greater quantities in PDA patient serum EVs compared to healthy subjects. The latter two were recently identified as a novel immune checkpoint, the PVR/nectin-2 T-cell immunoglobulin and ITIM domain (TIGIT) on T and NK cells [144], which when overexpressed led to NK cell dysfunction in acute myeloid leukemia [145]. This checkpoint could be a novel therapeutic target as anti-CTLA4, and anti-PD-1/PDL-1 therapies have found little success in PDA as compared with melanoma and non-small cell lung cancer. Overall, these authors demonstrated the involvement of tumor-derived EVs in modulating NK cells.

In an unconventional model of tumor cells derived-EV secretion and biomarker detection, having demonstrated that tumor growth far from the oral cavity can nonetheless impact secretion of EVs in the saliva, the authors used PDA mouse models to test the effects of saliva-EVs of tumor-bearing mice on healthy ones. The NK-cell activating receptors, CD69 and NKG2D, were downregulated, and the suppression of EV biogenesis confirmed that tumor cell derived-EVs were responsible for the effect of saliva-derived EVs on peripheral NK cell dysfunction. For the first time, it was hypothesized that tumor cells derived-EVs could systemically modulate the production of EVs from another gland, distant from the site of the tumor, and thus alter immune cell activity towards pro-tumoral [146]. However, the tumor EVs’ presence in the blood and lymphatic circulation cannot be eliminated to explain this phenomenon, and a comprehensive study of all tumor-derived EVs in systemic circulation and non-serum bodily fluids should be conducted to better understand this mechanism in order to give rise to the biomarker analyses.

Altogether, it is clear that EVs are involved in the modulation of innate and adaptive immune cells at all levels, highlighting the bidirectional, intricate connection between tumor and immune cells reinforcing the pro-tumoral rederivation of immune cells as well as the enhanced oncogenic abilities of PDA tumor cells.

### 3.5. The Impact of Tumor-Stroma EVs-Mediated Crosstalk on Metastatic Development and Niche Establishment

The major plight of patient survival with PDA can be largely attributed to a high metastatic burden. Aside from soluble factors such as chemokines and cytokines, as freely circulating entities within the organism, extracellular vesicles derived from PDA tumor cells have access to nearly every tissue and were found to target certain organs selectively, known as organotropism, to prime the formation of pre-metastatic niches. Thus, PDA tumor cells-derived EVs are modifying the microenvironment of these secondary sites, making the tissue capable of supporting tumor cell engraftment, survival and proliferation. PDA cells organotropism is mainly directed towards the liver, the peritoneum and the lungs [147]. It is now well understood that PDA tumor cells derived-EVs actively contribute to the preconditioning of these microenvironments and thus establishing the pre-metastatic niche. Using fluorescently-marked tumor-cell EVs in animal studies, it was found that the majority of circulating EVs ended up in the liver, where liver metastases were observed simultaneously. Interestingly, EVs were also found in the bone marrow, specifically in macrophages, and in Kupffer cells in the liver, indicative of a strong potential impact of PDA tumor cells derived-EVs dissemination on immune cell recruitment and the formation of an immunosuppressive microenvironment at the pre-metastatic niche [148]. Organ selection by EVs may be due to selective integrin expression, as was found in a large proteomics study of primary malignant PDA tumor cells derived-EVs content, revealing integrin beta-5 and differential integrin beta-4 expression for liver and lung organotropism, respectively, as well as the upregulation of pro-metastatic proteins such as annexin-A1, MET and S100A4 [149].

#### 3.5.1. Liver Metastases

A breakthrough study in the formation of liver metastases in PDA demonstrates the capability of PDA tumor cells derived-EVs to differentiate resident fibroblasts far from the primary tumor microenvironment. PDA tumor cells-derived EVs are selectively uptaken by tissue-resident macrophages of the liver, the Kupffer cells, which remodel the liver tissues via their effect on hepatic stellate cells. Altogether this sequential process ends up priming a metastatic niche that promotes the seeding of invasive tumor cells. These pro-metastatic EVs of PDA cancer cell lines and PDA patients’ sera upregulate macrophage migration inhibitory factor (MIF) as early as the formation of PanINs. MIF is normally involved in leucocyte recruitment and liver inflammation leading to fibrosis, but here, the authors demonstrated that MIF in PDA tumor cells derived-EVs targets KCs, stimulating their secretion of pro-inflammatory cytokine. Among them, the presence of TGFβ leads to hepatic stellate cell differentiation onto myofibroblasts. Those activated fibroblasts, as CAFs from the primary site, enhanced the deposition of fibronectin, mimicking the fibrotic microenvironment of the primary tumor microenvironment, which then promote the homing of bone-marrow-derived macrophages, indispensable for the formation of liver metastases. Interestingly, these steps occurred before the spontaneous formation of primary PDA lesions in mice, bringing into light the significance of PDA tumor cells derived-EV biomarker testing for early detection [150]. This study necessitates the inquiry of the sequential formation of an immunosuppressive environment via other immune cell populations in forming a pro-metastatic niche and collectively demonstrates the importance of the cross-talk between fibroblasts, their ECM remodeling capacity, and immune cells, all orchestrated by systemically circulating tumor-derived EVs, opening up new paths to therapeutic strategies.

EVs of highly PDA metastatic cells were found to increase amounts of activated HSCs, neutrophil recruitment in the liver and proportions of CD11^+^/GR1^+^ MDSCs in peripheral blood, resulting in increased primary tumor growth and higher metastatic burden when compared with EVs of lowly metastatic cells. As expected, concurrently, highly metastatic cell EVs received by other tumor cells increased their migration and invasion while reducing their adhesive properties. The internal cargo proteomics of highly metastatic vs. non-metastatic cells derived-EVs allowed to identify metabolic pathways, such as pyruvate, glycolysis and glutathione metabolism, indicative of a crosstalk via EVs, allowing future cancer cells to seed in the microenvironment, following metabolic reprogramming, to support the hostile pre-metastatic niche [151].

#### 3.5.2. Lung Metastases

In lung metastases, the involvement of an isoform of protein kinase D, protein kinase D-1, was identified as a regulator of PDA tumor cells derived-EVs content and subsequently lung metastatic niche formation and burden. PRKD1 was found to be downregulated in other cancers as it inhibits cancer cell motility via its impact on actin. In PDA, a subgroup of PDA tumor cells demonstrates PRKD1 downregulation or inhibition together with increased EV secretion. Indeed, via reduced phosphorylation of PRKD1 substrate cortactin, PRKD1^−^ PDA tumor cells have an enhanced branching of actin filaments facilitating EVs loading and secretion. These EVs of cells with low PRKD1 contained higher amounts of invasiveness-associated proteins, such as tenascin C, and expressed integrin α6β4, specifically targeting EVs to the lung and promoting a pre-metastatic niche formation. Unsurprisingly, in this case, integrin β5, targeting the liver, was found to be downregulated. Lung fibroblasts following PDA tumor cells derived-EVs treatment were activated with an α-SMA^+^ phenotype and highly expressed S100A proteins linked to fibrosis and poor survival. This study identified a novel phenotype of PDA, also present in humans, based on PRKD1 expression and its impact on the quantity and characteristics of EVs, which in turn are responsible for priming lung pre-metastatic niches [152]. This lung organotropism of metastatic PDA tumor cells was further investigated by Ogawa et al., demonstrating that prometastatic secretome trafficking, via exosomes, initiates pancreatic cancer pulmonary metastasis through the ASPH-Notch axis [153].

Other potential targets in PDA tumor cells derived-EVs include tetraspanins CD151 and Tspan8, which were found to impact several aspects of lung niches, including immune cells, fibroblasts, matrix and EMT. These tetraspanins were responsible for matrix degradation to support secondary tumor cell adhesion motility, induce a pro-inflammatory response from hematopoietic cells, such as SDF1, CXCR4, VEGFR1, impacting lymphatic angiogenesis, and most importantly upregulated Notch and thus induced EMT in non-metastasizing cells. Overall, metastatic lung burden was largely influenced by EVs containing CD151 and Tspan8, demonstrating their relevance in promoting metastases from several aspects [154].

## 4. Conclusions

**Figure 1:**
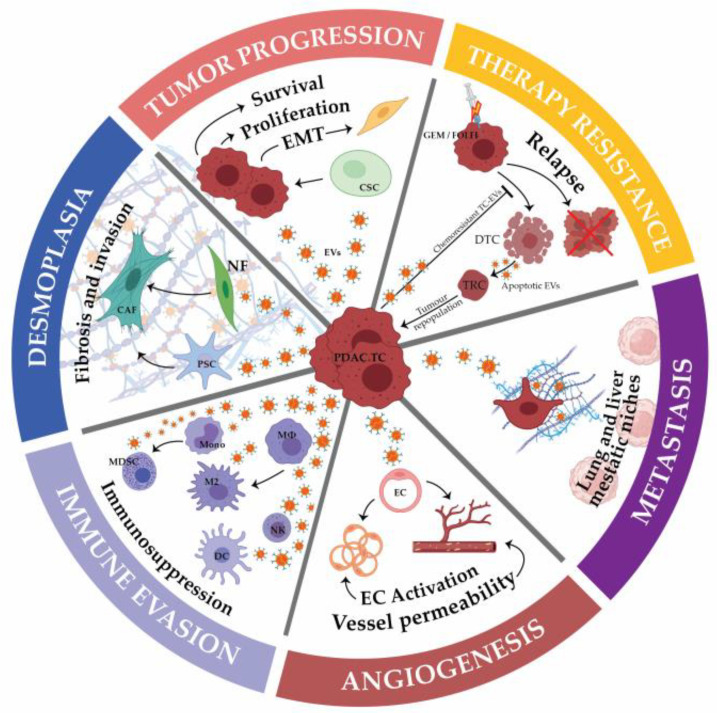
Schematic representation of tumor–stromal cell intercellular communication via EVs and its subsequent outcomes on major cancer hallmarks in PDAC. Cell types: TCs—tumor cells, NFs—normal fibroblasts, PSCs—pancreatic stellate cells, CAFs—cancer-associated fibroblasts, CSCs—cancer stem cells, DTCs—dying tumor cells, TRCs—tumor repopulating cells, GEM—gemcitabine, FOLFI—folfirinox, ECs—endothelial cells, Mφ—macrophages, M2—M2 macrophages, Mono—monocyte, MDSC—myeloid-derived suppressor cells, DCs—dendritic cells, NK—natural killer cells.

In this review, we described the various communication lines established by EVs secretion and reception between PDA tumor cells and stromal cells and the importance of these intercellular communication mediators in PDA tumorigenesis in priming of secondary tumor sites for metastases development and for therapy resistance (Figure 1). With regards to such recently uncovered yet significant evidence, it is clear that deciphering in detail an EV network in PDA would offer new avenues for therapeutic options, even besides the use of EVs as therapeutic vectors, which is undoubtedly an expanding field. Building on preceding knowledge of EVs’ implication in tumor advancement, uncovering the implication of host body-EVs, such as ones from cells of metastatic niches or bone-marrow-derived mesenchymal cells, would further strengthen and broaden therapeutic options, allowing to halt therapeutic resistance mechanisms. However, while the field of research of EVs is rapidly growing and with technical applications under development, the studies involving EVs must be standardized in extracellular vesicle isolation methods, cancer cell lines and -omics analyses to determine which of the numerous molecules contained within EVs directs malignant crosstalk and whether they act cooperatively. The future research aiming at targeting PDA tumor cells should systematically integrate this cellular component, considering it as a more complex entity, as EVs-driven intercellular dialogue represents a unique way to deliver multifarious messages simultaneously.

## Figures and Tables

**Table 1:** Tumor to tumor cell crosstalk mediated by EVs.

Donor Cells	Recipient Cells	Functional Cargo	Implication in PDAC	PMID Reference
TCs	TCs	miR-192-5p	Overexpressed in plasma/serum exosomes; Diagnostic capacity comparable to CA-19	32630552
		miR-23b-3p	Upregulated in serum and PANC-1 supernatant exosomes; Prognostic value close to CA-19	28849236
		lncRNA NONHSAT105177	Overexpressed in TCs EVs when treated with mettilin, known to increase chemosensitivity	30237397
		Asparaginyl endopeptidase	Overexpression associated with reduced patient survival; Found in serum exosomes	29568945
Highly malignant TCs	Low malignant TCs	circRNA PDE8A	Overexpressed in liver metastases EVs, plasma exosomes; Associated with poor survival	29709702
		Zinc finger protein 4	Associated with tumour growth and reduced survival	30007115
TCs	Cancer stem cells	Ceramide-1-phosphate	NR	30222969
Invasive TCs	Non invasive TCs	miRNA-222	Overexpressed in plasma and invasive TCs’ exosomes; Correlates with survival	30458449
		lncRNA Sox2ot	Overexpressed in patient plasma EVs; Associated with low survival and aggressiveness	29643475
Metastatic TCs	Non metastatic TCs	miRNA-339-5p	Downregulated in highly metastatic TCs’ exosomes	31588120
		miRNA-125b-5p	Upregulated in invasive TCs exosomes; Associated with metastases	32470446
Cancer-initiating cell	Non cancer-initiating cell	CD44v6	Metastases promotion	27419629
		Claudin-7	NR	30945750
Gemcitabine-resistant TCs	Gemcitabine-sensitive TCs	miR-210	NR	31713003
		miR-155	Overexpression associated with reduced patient survival	28198398, 28152544
		Ephrin type-A receptor 2	NR	30613276
Dying TCs	Repopulating TCs	miR-194-5p	Associated with peritoneal recurrence	32228703

EVs—extracellular vesicles, TCs—tumor cells, NR—Not Reported.

**Table 2:** Tumor cell and stromal cell crosstalk mediated by EVs.

Donor Cell	Recipient Cell	Functional Cargo	Implication in PDAC	PMID Reference
TCs	Fibroblasts	TGFb	NR	21098712
		Annexin A1	Acquisition of mesenchymal phenotype and metastases	33353163
		hTERT mRNA	NR	27385095
TCs (with GOF p53 mutation)	Fibroblasts	Podocaxylin (PODXL)	NR	30498210
TCs	PSCs	miR-1260	NR	29111329
		Lin28B	NR	31413760
PSCs	TCs	miR-451a	NR	27841793
		miR-5703	NR	32585413
		miR-21	NR	32319558
Hypoxic PSCs	TCs	miR-4465, miR-616-3p	NR	33653966
CAFs	TCs	Annexin-6	ANXA6+ EVs correlate with poor patient survival and metastases	27701147
CAFs	TCs	Snail mRNA, miR-146a	NR	27669441
		miR-106b	NR	31374207
TCs	Lung fibroblasts	a6B1, a6b4	NR	32446697
TCs	ECs	Tetraspanin-8	NR	16849554, 20124479
		Circ-IARS	Correlated with liver metastases, vascular invasion and TNM stage	30064461
		Tissue factor	NR	29164060, 32006891
		Annexin A1	Acquisition of mesenchymal phenotype; Increased metastases	30518142
		Myoferlin	NR	27845903
		miR-27	NR	31724333
Hypoxic TCs	ECs	UCA1	Overexpressed in patient serum, correlated with poor survival	32942233
TCs	Fibroblasts, ECs, HCs	Tetraspanin-8 & CD151	NR	25544774
TCs	Adipocytes	Adrenomedullin	Early onset weight loss and diabetes in PC patients	6061593
TCs	Monocytes	CDD4H	NR	30957871
		Hyaluronan	NR	26210045
		Receptor tyrosine kinases	NR	26895960
Hypoxic TCs	Macrophages	miR-301a-3p	Patient serum exosome associated with invasion and poor prognosis	29880482
TCs	Macrophages	Ezrin	High-Ezrin+ EVs in patient blood associated with shorter OS	32064151
TCs	Kupffer cells	MIF	Plasma level correlated with liver metastases	25985394
M2 Macrophages	TCs	lncRNA SBF2-AS1	NR	32301277
		miR-501-3p	Found highly expressed in human PDAC tissues	31307515
		miR-365	NR	30042153
		Chitinase-3-like-1, Fibronectin	Associated with decreased OS and low response to therapy	33212158
	CSCs	miR-21a-5p	NR	33728488
TCs	Dendritic cells	miR-203	NR	25290620
		miR-212-3p	NR	26337469
SMAD4+ TCs	MDSCs	hsa-miR-1260a, glycolytic enzymes	NR	29156694
TCs	Mast cells	CD73	NR	31493676
TCs	NK cells	TGFb1; miR23a	NR	27141372
NK cells	TCs	miR-3607-3p	Downregulated in plasma Evs; Predict poor prognosis	31921112

EVs—extracellular vesicles, TC—tumor cells, PSC—Pancreatic Stellate cells, CAFs—cancer-associated fibroblasts, ECs—Endothelial cells, HCs—Haematopoietic cells, CSC—cancer stem cells, MDSC—Myeloid-Derived Suppressor cells, NK—Natural Killer, OS—overall survival.

## Data Availability

No new data were created or analyzed in this study. Data sharing is not applicable to this article.
